# Direct Measurement of Oxygen Mass Transport at the Nanoscale

**DOI:** 10.1002/adma.202105622

**Published:** 2021-10-05

**Authors:** Federico Baiutti, Francesco Chiabrera, David Diercks, Andrea Cavallaro, Lluís Yedra, Lluís López‐Conesa, Sonia Estradé, Francesca Peiró, Alex Morata, Ainara Aguadero, Albert Tarancón

**Affiliations:** ^1^ Department of Advanced Materials for Energy Catalonia Institute for Energy Research (IREC) Jardin de les Dones de Negre 1 Sant Adrià de Besòs (Barcelona) 08930 Spain; ^2^ Department of Materials Chemistry National Institute of Chemistry Hajdrihova 19 Ljubljana SI‐1000 Slovenia; ^3^ Department of Energy Conversion and Storage Functional Oxides group Technical University of Denmark Fysikvej, 310 Kongens Lyngby 233 2800 Denmark; ^4^ Department of Metallurgical and Materials Engineering Colorado School of Mines Golden CO 80401 USA; ^5^ Department of Materials Imperial College London Prince Consort Road London SW7 2BP UK; ^6^ Laboratory of Electron Nanoscopies (LENS) Micro‐Nanotechnology and Nanoscopies for electrophotonic Devices (MIND) Department of Electronics and Biomedical Engineering and Institute of Nanoscience and Nanotechnology (IN2UB) University of Barcelona C/Martí i Franquès 1 Barcelona 08028 Spain; ^7^ TEM‐MAT Unit Scientific and Technological Centers of the University of Barcelona (CCiTUB) C/Lluís Solé i Sabaris 1 Barcelona 08028 Spain; ^8^ ICREA Passeig Lluís Companys 23 Barcelona 08010 Spain

**Keywords:** electrode materials, fuel cells, grain boundaries, mixed ionic‐electronic conductors, oxygen kinetics, thin films

## Abstract

Tuning oxygen mass transport properties at the nanoscale offers a promising approach for developing high performing energy materials. A number of strategies for engineering interfaces with enhanced oxygen diffusivity and surface exchange have been proposed. However, the origin and the magnitude of such local effects remain largely undisclosed to date due to the lack of direct measurement tools with sufficient resolution. In this work, atom probe tomography with sub‐nanometer resolution is used to study oxygen mass transport on oxygen‐isotope exchanged thin films of lanthanum chromite. A direct 3D visualization of nanoscaled highly conducting oxygen incorporation pathways along grain boundaries, with reliable quantification of the oxygen kinetic parameters and correlative link to local chemistries, is presented. Combined with finite element simulations of the exact nanostructure, isotope exchange‐atom probe tomography allowed quantifying an enhancement in the grain boundary oxygen diffusivity and in the surface exchange coefficient of lanthanum chromite of about 4 and 3 orders of magnitude, respectively, compared to the bulk. This remarkable increase of the oxygen kinetics in an interface‐dominated material is unambiguously attributed to grain boundary conduction highways thanks to the use of a powerful technique that can be straightforwardly extended to the study of currently inaccessible multiple nanoscale mass transport phenomena.

## Graphical abstract

A novel method based on atom probe tomography for the direct measurement of ion kinetics at the nanoscale is reported. Extremely high 3D resolution (<1 nm) and straight correlation with local cation distribution are demonstrated for the exemplary case of a mixed ionic–electronic conductor exhibiting enhanced grain boundary oxygen diffusivity.

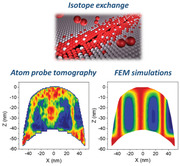

## 1 Introduction

Ion migration is the underlying mechanism governing the performance of a collection of cornerstone solid.state energy devices such as batteries, fuel cells, and electrolyzers.^[^ ^1^, ^2^, ^3^ ^]^ The search for enhanced mass transport kinetics has, in recent years, brought to the fore the use of engineered interfaces, which may promote nanoscale effects to potentially dominate over the material's bulk behavior.^[^ ^4^ ^]^ Such local phenomena include the formation of space‐charge layers,^[^ ^5^, ^6^ ^]^ strain‐induced mobility, concentration effects,^[^ ^7^, ^8^, ^9^, ^10^ ^]^ or cationic local non‐stoichiometries.^[^ ^11^ ^]^ Striking examples of local fast ionic conduction have been reported recently: For example, grain‐boundary (GB) nanostructuring has been shown capable of turning a mainly electronic conductor such as La_0.8_Sr_0.2_MnO_3_ into a mixed ionic‐electronic conductor (MIEC) with remarkable oxygen kinetics.^[^ ^7^ ^]^ This effect has been ascribed to the maximized density of fast conducting grain boundaries with orders of magnitude enhanced oxygen mass transport properties.^[^ ^12^ ^]^ Mesoscopic systems such as superlattices,^[^ ^13^ ^]^ vertically aligned nanocomposites (VANs),^[^ ^14^, ^15^ ^]^ or nanocrystalline materials offer therefore an enormous potential for the development of ad hoc high‐performing materials for energy applications. However, the deployment of strategies for increasing mass transport at the interface level is often hindered by a lack of understanding of the fundamental controlling mechanisms, which remain largely undisclosed to date.^[^ ^11^, ^16^ ^]^

Several techniques based on electrical conductivity relaxation,^[^ ^17^ ^]^ weight changes,^[^ ^18^ ^]^ electrochemical impedance spectroscopy,^[^ ^19^ ^]^ and color changes,^[^ ^20^, ^21^, ^22^ ^]^ are widely employed in order to retrieve global information on ion transport in oxides, but are insufficient for the study of local kinetics at the nanoscale. The most common technique for the investigation of ion transport properties (oxygen diffusivity—*D*—and surface exchange coefficient—*k*) is isotopic exchange depth profiling (IEDP) in combination with secondary‐ion mass spectrometry (SIMS).^[^ ^23^, ^24^, ^25^ ^]^ However, due to a limited lateral resolution of the IEPD‐SIMS technique (>100 nm),^[^ ^26^ ^]^ a detailed spatial picture of the ionic tracer diffusion profile is not achievable. The introduction of methodologies capable of directly observing mass transport phenomena with nanometer resolution is desired.

Excitingly, atom probe tomography (APT) has recently been presented as an alternative technique for obtaining quantitative 3D compositional maps with sub‐nanometer spatial resolution and tens of parts‐per‐million concentration sensitivity,^[^ ^27^ ^]^ offering a detailed picture of the cationic and anionic distributions along with isotopic sensitivity.^[^ ^28^, ^29^, ^30^, ^31^ ^]^ Such a technique, therefore, has the potential for offering new insights into the structure–function relationship at the nanoscale. In the present article, we use atom probe tomography to study the GBs of nanocrystalline thin films of lanthanum chromite (La_0.9_Sr_0.1_CrO_3_—LSCr), previously exchanged with ^18^O. Through this isotopic‐exchange APT (IE‐APT) we demonstrate that GBs in LSCr provide local ultrafast oxygen exchange pathways and we map the singularities of such preferential highways—namely GB geometry and anionic and cationic local distributions—with 3D nanometer‐resolution. We show that via a simple thin‐film nanostructuring approach, one can achieve a local enhancement of oxygen kinetics in LSCr of ≈4 orders of magnitude in *D* and ≈3 orders of magnitude in *k*. These results in remarkable absolute values of $D_{\text{gb}}^{*}=6.5\;\times \;10^{-12}$ cm^2^ s^–1^ and$k_{\text{gb}}^{*}=9.5\;\times \;10^{-8}$ cm s^–1^ at 640 °C and converts a mainly electronic conductor with very poor oxygen kinetics under oxidizing atmospheres such as LSCr^[^ ^32^ ^]^ into an excellent MIEC, whose local surface exchange and mass transport properties can compete with the best electrode materials employed in solid oxide cells.^[^ ^33^, ^34^ ^]^

## 2 Results and Discussion

**Figure 1:**
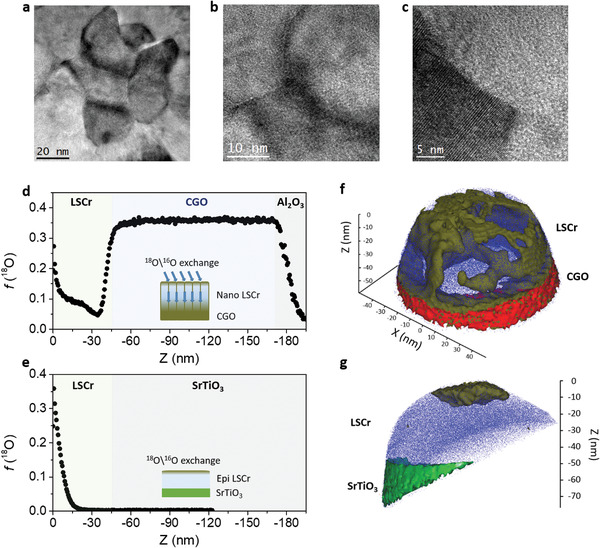
Characterization of nanocrystalline and epitxial thin films of LSCr. a–c) Top‐view HR‐TEM of nanocrsytalline LSCr at different magnifications. d,e) Depth profiles of ^18^O fraction measured by IEDP‐SIMS for a nanocrystalline LSCr thin film deposited on 130 nm CGO/Al_2_O_3_ (0001) (d) and an epitaxial LSCr layer on SrTiO_3_ (001) (e). f,g) 3D ^18^O fraction measured by IE‐APT for the nanocrystalline (f) and epitaxial (g) layers. The golden‐brown isoconcentration surfaces depict regions containing at least 10 at% ^18^O. The blue dots show a fraction of the Cr cationic species. The red (green) isoconcentration surfaces demarcate the bottom layers (CGO in (f) and SrTiO_3_ in (g), respectively).

In order to unveil the grain boundary effect on the mass transport properties of LSCr, we fabricated nanocrystalline and epitaxial thin films by pulsed laser deposition (thickness ≈ 50 nm). Nanocrystalline films were deposited on top of Al_2_O_3_ single crystal substrates and comprise a 130 nm‐thick intermediate layer of Ce_0.8_Gd_0.2_O_2_ (CGO), while epitaxial films were fabricated directly on top of SrTiO_3_ single crystals. In **Figure** **1**a–c, planar view high‐resolution transmission electron microscopy (HR‐TEM) images of nanocrystalline LSCr are shown. The layer appears as fully dense and free from intergranular secondary phases. The average grain size is 34 nm. Please refer to Note S1, Supporting Information, for additional TEM characterization, together with high‐resolution X‐ray diffraction (HR‐XRD) and atomic force microscopy (AFM). Please refer to Figure S2, Supporting Information, for structural characterization of epitaxial LSCr, highlighting the full coherence between substrate and film according to HR‐XRD and related reciprocal space mapping (high‐quality epitaxy).

Traceable oxygen diffusion profiles were created by high‐temperature annealing in enriched atmosphere (≈90% ^18^O_2_, 1800 s, 640 °C—see sketch in the inset of Figures 1d,e for nanocrystalline and epitaxial LSCr, respectively). The resulting depth profiles of the ^18^O isotope fraction (*f* 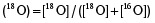), as retrieved by conventional ToF‐SIMS—that is, averaging a surface area of 50 µm × 50 µm—are presented in Figures 1d,e for the nanocrystalline and the epitaxial layers, respectively. The two profiles exhibit a striking difference. In the case of the GB‐free epitaxial material (cf. Figure 1e), the penetration length of the oxygen isotopes is limited to the surface region (<15 nm), with a decay profile which can be satisfactorily fitted by Crank solution to Fick's law (see Note S2, Supporting Information, for more details). This is the expected bulk behavior of LSCr.^[^ ^32^ ^]^ Introducing grain boundaries (cf. Figure 1d) results in a much deeper penetration of the tracer oxygen and the formation of additional features in the isotope profile, namely, a deep tail for *z* > 10 nm in LSCr and a step at the LSCr/CGO interface. The tracer concentration in the buffer layer of the oxide‐ion conductive CGO bottom layer is constant at high values (≈35%). The distinctive features described for the IEDP‐SIMS profile in Figure 1d suggest preferential ^18^O diffusion through GBs;^[^ ^35^ ^]^ however, alternative explanations, such as presence of nano‐ (micro)‐voids and cracks and surface space‐charge layer effects, potentially giving rise to a similar profile, cannot be ruled out.^[^ ^36^ ^]^ Even more importantly, this measurement method conceals important local information on grain and GB oxygen isotope concentration—as generally IEDP‐SIMS is not capable of achieving sufficient resolution in 3D—and is not structure‐sensitve.^[^ ^37^ ^]^ Only average values of the ^18^O fraction and of chemical composition, obtained over a large lateral area, are obtained (here 50 µm × 50 µm). In such a situation, the retrieval of local kinetic parameters requires the application of simplified models,^[^ ^7^, ^35^ ^]^ for which a number of a priori assumptions have to be made (e.g., grain geometry, average grain size, extent of the GB region). Such an approach has severe implications on the reliability of the resulting kinetic parameters, which are characterized by great uncertainty even for the same material.^[^ ^38^ ^]^ Conversely, IE‐APT provides a unique set of information and a much more unambiguous picture on the local mass diffusivity and on the chemistry of nanostructures with sub‐nm 3D resolution. This is shown in Figures 1f,g with respect to oxygen transport properties (and quantified later in the text), where 3D ^18^O isoconcentration surfaces for the very same nanocrystalline and epitaxial samples, respectively, are reported. The IE‐APT analysis of the nanocrystalline LSCr (please refer also to Video S1, Supporting Information, for a top‐view slicing), directly highlights the presence of preferential mass diffusion pathways which are almost exclusively confined in narrow regions (nanometer‐wide). Such high $f{(}^{18}\text{O})$ concentration areas are well‐aligned along the growth direction and are comparable in size to the GBs as retrieved by TEM (cf. Figure 1a). A much lower ^18^O content is present outside of these regions (grain bulk). Conversely in the case of the GB‐free epitaxial material (Figure 1g), the layer shows no preferential pathway for ^18^O but an accumulation near the surface region, confirming the poor diffusivity of bulk LSCr. To the best of the authors’ knowledge, high oxygen diffusion in LSCr has not been reported previously, with only early work on enhanced grain boundary diffusion in a related nanocrystalline bulk material (La,Ca)CrO_3_.^[^ ^39^ ^]^

**Figure 2:**
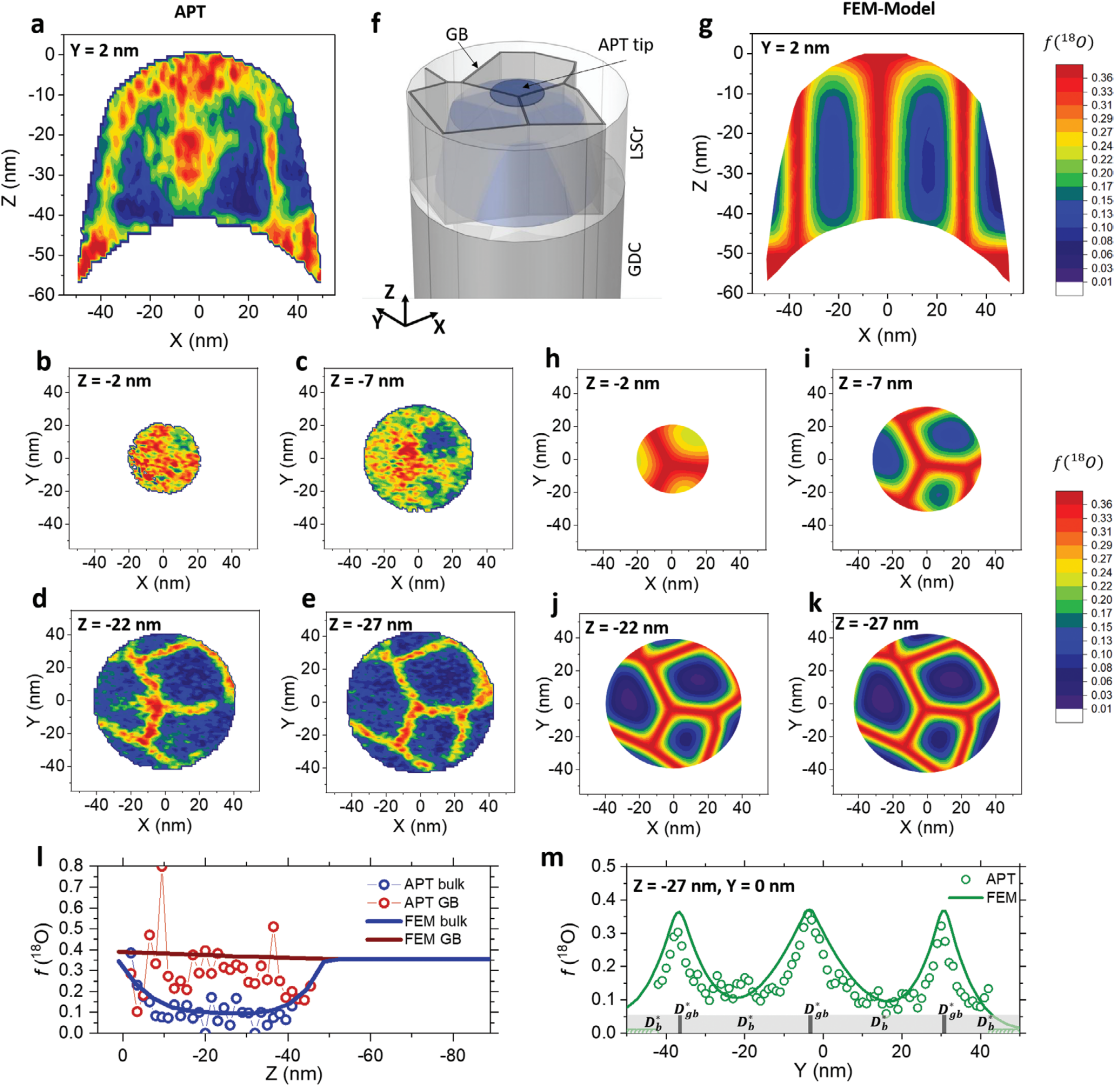
Modeling ^18^O transport in LSCr GBs. a) Vertical and b–e) horizontal (taken at different distances from the free surface) cross sections of the ^18^O fraction measured by IE‐APT. f) Schematic representation of the APT tip superimposed within the 3D FEM geometry. g–k) Calculated vertical (g) and horizontal (h–k) FEM simulations. l) 1D depth profiles of the oxygen fraction measured by APT along a GB (red dots) and in the grain bulk (blue dots). The 1D depth profiles obtained by FEM simulations along the same cutting lines are also shown in (l) as continuous lines. m) 1D ^18^O fraction along the *y*‐direction obtained at *Z* = −27 nm and *X* = 0 nm by IE‐APT (open dots) and FEM modeling (green line). At the bottom of (m), the width of the areas modeled using D*_b_ and D*_gb_ are shown.

Such 3D reconstructions can be assessed quantitatively in a straightforward manner, as shown in **Figure** **2**. Here, high‐resolution 2D ^18^O concentration profiles, obtained by IE‐APT, are represented by vertical and horizontal 2D contour plots for nanocrystalline LSCr. These were obtained by integration of 2.5 nm‐wide rectangular regions of interest (ROIs) at different positions along the APT tip reconstruction (out‐of‐plane at fixed *y =* 2 nm in Figure 2a and in‐plane at fixed *z* values of −2, −7, −22 and −27 nm in Figures 2b–e, respectively). Such cross‐section profiles give a clear visualization of the fast oxygen transport along the LSCr GBs and offer the possibility of a precise quantification of the diffusion profiles. As shown in Figure 2a, IE‐APT provides an unprecedented picture of the oxygen local pathways: ^18^O is incorporated through the GBs at the surface of LSCr, progressing toward the CGO buffer layer (bottom of the vertical section in Figure 2a) along the grain interfaces. It becomes apparent that nanocrystalline LSCr is characterized by ≈35 nm‐separated fast oxygen diffusion channels in which 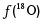 ≈ 0.35 (cf. Figure 2a). Such “oxygen highways” are well‐defined especially for *z* < −20 nm and are ≈10 nm wide, whereas the top‐region (closer to the surface) is generally ^18^O‐enriched (cf. Figure 2b–e). Please note that the geometry and grain size defined by the fast diffusion channels are consistent with the grain size as resulting from TEM analysis—cf. Figure 1.

In order to retrieve the relevant oxygen mass transport parameters, that is, *D* and *k*, the obtained experimental ensemble has been translated into a 3D finite element model (FEM) for simulations of an equivalent geometry. Figure 2f shows a schematic representation of the APT tip superimposed within the 3D geometry adopted in the FEM simulation. The geometry consists of columnar‐like grains of LSCr (≈35 nm wide) on top of a 130 nm‐thick CGO layer which, under the exchange conditions, acts as a reservoir of oxygen due to its high diffusivity. Note that, thanks to the APT 3D resolution, one can build an accurate FEM model whose grain size and shape is the almost exact schematization of the experimental data. Time dependent Fick's second law was solved considering two different oxygen kinetics parameters for the diffusivity and the oxygen incorporation in bulk and GBs (*k*_gb_, *D*_gb_ and *k*_b_, *D*_b_ for GBs and bulk, respectively). The simulations were optimized by systematic parametrization of the oxygen transport magnitudes (more details about the FEM simulations can be found in Note S3, Supporting Information). The results of the FEM analysis are reported in Figure 2g (vertical contour) and Figure 2h–k (horizontal contours), in direct comparison with the experimental data taken for the same cuts. The results of the simulations offer an excellent fit to the 3D experimental data, highlighting the powerful approach of the method. This is also confirmed by complementary 1D out‐of‐plane and in‐plane profiles (retrieved from 1 nm‐wide cylindrical ROIs) presented in Figures 2l,m, respectively (see also Figure S7, Supporting Information, for additional linescans). From this analysis, a very high local diffusivity toward the CGO buffer layer is confirmed only along the GB with a very low ^18^O concentration gradient measured in the off‐plane direction (red open symbols in Figure 2l). The oxygen tracer is also observed to diffuse radially from the GBs toward the grain bulk, giving rise to an in‐plane profile that can be used to precisely determine D*_b_, as seen in Figure 2m. Unlike SIMS data,^[^ ^7^, ^12^ ^]^ we highlight here that APT provides direct, independent information on local oxygen concentration of bulk and GB—with near‐atomic 3D spatial resolution—allowing drastic improvement of the quality and the confidence of the FEM numerical model. This is exemplified in Figure S8, Supporting Information, where we show that IEDP‐SIMS depth profiles can be satisfactorily fit by two very different sets of oxygen transport parameters, one similar to the IE‐APT results (therefore validating the presented method) and the other leading to an unrealistically high ^18^O fraction in the GB's region. The possibility of such a misleading SIMS data interpretation is resolved by using the IE‐APT method. As an additional information on this powerful methodology, we report here that the measurement of one sample by APT took, in our case, between 3 to 5 h including initial preparation of a tip batch (five tips for each sample). The best three tip specimens for each sample were analyzed by APT, with a success rate >80%. Please refer to Section 4 for further details on sample preparation.

**Figure 3:**
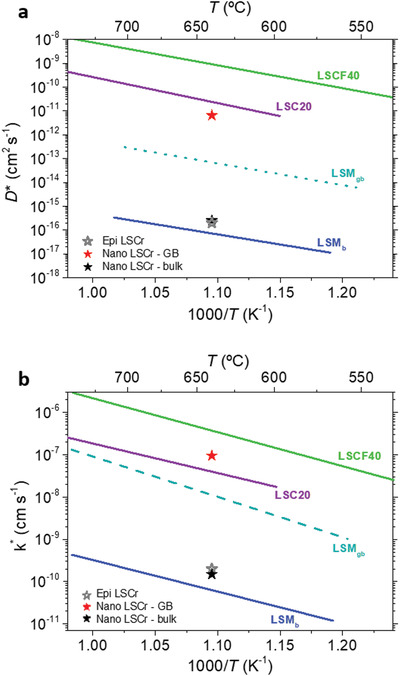
Oxygen mass transport properties in LSCr bulk and GBs. a) Oxygen diffusivity and b) surface exchange coefficient of bulk and GBs of LSCr obtained by IE‐APT. Bulk values extracted from IEDP‐SIMS analysis of epitaxial LSCr layers are also shown. A set of literature data for La_0.8_Sr_0.2_MnO_3±δ_ (LSM) bulk and GBs,^[^ ^7^ ^]^ La_0.8_Sr_0.2_CoO_3±δ_ (LSC20) bulk^[^ ^40^ ^]^ and La_0.8_Sr_0.4_Co_0.4_Fe_0.6_O_3±δ_ (LSCF40)^[^ ^42^ ^]^ are also reported for comparison.

The final oxygen transport parameters obtained by the IE‐APT model fitting for our nanocrystalline LSCr layer highlight over 4 orders of magnitude enhancement of the diffusivity (D*_b_ = 2.5 × 10^−16^ cm^2^ s^–1^, D*_gb_ = 6.5 × 10^−12^ cm^2^ s^–1^) and ≈3 orders of magnitude increase in surface exchange (k*_b_ = 1.5 × 10^−10^ cm s^–1^ and k*_gb_ = 9.5 × 10^−8^ cm s^–1^) with respect to the bulk at 640 °C. Such *k*‐ and *D*‐values are compared in **Figures** **3**a (*D*) and 3b (*k*), respectively, to the ones resulting from conventional SIMS measurements for epitaxial LSCr (Note S2, Supporting Information) and to relevant electrode materials for Solid Oxide Cells applications. One can observe that the bulk kinetic parameters obtained by IE‐APT on the nanocrystalline samples are very similar to the results of the SIMS analysis on epitaxial LSCr. This proves the robustness of the nanoscale APT‐based method and demonstrates that the enhanced oxygen transport properties observed in the nanocrystalline thin films originate entirely by the presence of GBs. On the other hand, comparison with the state‐of‐the‐art electrode materials shows that the observed nanoscale enhancement converts LSCr into an MIEC material having local properties comparable to La_0.8_Sr_0.2_CoO_3±δ_,^[^ ^40^ ^]^ that is, nanocrystalline LSCr completely changes its nature from a mainly electronic conductor to a good MIEC with remarkable oxygen surface exchange properties. Although enhanced oxygen vacancy (${\text{V}}_{\text{O}}^{\cdot \cdot }$) concentration and mobility—which is inferred here by the measured local fast oxygen kinetics—is expected at GBs of donor‐doped perovskites,^[^ ^41^ ^]^ it should be noted here that the increase of oxygen diffusivity and surface exchange in LSCr is remarkably larger than the one previously reported at the GBs of La_0.8_Sr_0.2_MnO_3±δ_, which is considered the seminal example of a GB‐dominated MIEC material.^[^ ^7^ ^]^

**Figure 4:**
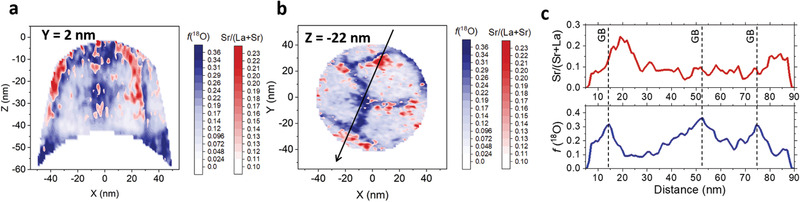
Sr segregation in LSCr thin films. a) Vertical and b) horizontal cross sections of the relative Sr accumulation in nanocrystalline LSCr thin films. The oxygen fraction maps are also overlayed in (a,b) to highlight the region of fast oxygen diffusion. c) 1D concentration profiles of Sr/(Sr + La) and f(^18^O) measured along the black line shown in (b). The vertical dashed black lines highlight the positions of the oxygen fast diffusion regions (GB).

As previously outlined by Haile and co‐workers,^[^ ^29^ ^]^ a very interesting feature of the APT analysis is the possibility of mapping both cationic and anionic species at the same time with nanometer‐resolution, thus drawing a comprehensive 3D chemical picture of great importance for electrical and electrochemical properties.^[^ ^28^, ^29^ ^]^ This is enhanced in IE‐APT since also oxygen isotopes can be superimposed to cation and anion distributions. Our particular focus in this work is on the distribution of Sr in the ^18^O‐exchanged nanocrystalline LSCr films. Note also that a slight Cr depletion in the grain interior, possibly as a cause of global off‐stoichiometry or of Cr segregation at the GBs,^[^ ^43^, ^44^ ^]^ was observed (Figure S9, Supporting Information). **Figure** **4** shows the Sr relative concentration contour plots (i.e., Sr/(Sr + La)) superimposed onto the ^18^O fraction, both measured by APT and analyzed based on 2.5 nm‐wide in‐plane ROIs. Please refer to Figure S10, Supporting Information, for a full characterization (including additional in‐plane ROIs) for ^18^O fraction, Sr fraction, and total oxygen distribution. In Figure S10, Supporting Information, note that, possibly as a cause of slight trajectory aberration at phase boundaries,^[^ ^45^ ^]^ the expected oxygen deficiency at the GB cannot be directly observed and that the ^18^O‐rich areas should be considered as a fingerprint for mobile oxygen vacancies (${\text{V}}_{\text{O}}^{\cdot \cdot }$) accumulation.^[^ ^11^, ^28^ ^]^ The slice analysis in Figure 4 indicates strong Sr segregation in the vicinity of the fast oxygen diffusion pathways. Such a Sr demixing leads to the formation of narrow, partially interconnected, high dopant concentration volumes of a few nanometers in width that run along—yet not fully overlapping with—the fast oxygen diffusion regions (see Figures 4a,b for out‐of‐plane and in‐plane contours, respectively). It is noteworthy that no secondary phase precipitates could be observed by TEM (cf. Figure 1) and that the La concentration is complementary to Sr (cf. Figure S11, Supporting Information), that is, Sr is present a substitutional cation in the LSCr sublattice (${\text{Sr}}_{\text{La}}^{/}$). The selected linescan in Figure 4c allows for an easy quantification of the Sr concentration maximum (up to ≈0.25), while the bulk value is close to the nominal (0.1). It is confirmed here that Sr accumulates around some of the ^18^O high‐concentration regions only, that is, GBs in LSCr present a highly inhomogeneous composition. Segregation of dopant toward the interfaces and to the free surface (cf. Figure S12, Supporting Information, for the Sr concentration integrated over a vertical contour) is a well‐known phenomenon stemming from the combination of elastic and electrostatic effects.^[^ ^41^, ^46^, ^47^ ^]^ It is also known that GBs provide a fast cation diffusion pathway for Sr segregation toward the surface and for the sequent formation of highly insulating SrO,^[^ ^43^ ^]^ one of the main causes of performance degradation in high‐temperature electrode materials.^[^ ^48^ ^]^ Here, the Sr concentration gradient caused by interface segregation can be quantified in width (≈10 nm across the GBs) and concentration by APT (cf. Figure 4c). Moreover, the spatial relationship between Sr‐ and mobile ${\text{V}}_{\text{O}}^{\cdot \cdot }$‐accumulation areas can be disclosed. Our analysis points out that such zones are spatially separated at the nanoscale in LSCr, ruling out a bulk‐like electroneutral situation. Rather, based on the measured distribution of ionic defects, local electric field should be considered in agreement with a space‐charge scenario in which ${\text{V}}_{\text{O}}^{\cdot \cdot }$ accumulation zones are compensated by spatially separated, negatively charged ${\text{Sr}}_{\text{La}}^{/}$.^[^ ^49^ ^]^ Note that Sr–La interdiffusion is expected to be active during preparation and post‐annealing and that also a decrease in the hole concentration is predicted.^[^ ^43^, ^50^, ^51^, ^52^ ^]^ Unlike currently employed models however—which stem from symmetric boundary conditions and result in a symmetric response on either side of the GB—here Sr‐accumulation regions are discontinuous and asymmetric, that is, GBs are strongly inhomogeneous. Such a finding suggests that additional structural parameters—possibly modifying the local thermodynamic equilibrium by introducing an elastic energy term—should be taken into account for a full description of randomly oriented interfaces in nanocrystalline LSCr (chemo‐mechanical coupling).^[^ ^53^ ^]^

## 3 Conclusion

We have directly observed oxygen fast diffusion pathways along grain boundaries in a mainly electronic conductor, LSCr, using atom probe tomography in oxygen‐isotope exchanged nanocrystalline samples. Bulk and grain boundary oxygen ion diffusivities and surface exchange coefficients are quantified with remarkable detail and exhibit a local enhancement of four and three orders of magnitude, respectively, converting LSCr into a good mixed ionic‐electronic conductor with excellent oxygen exchange properties. This highlights the power of nanostructuring for the fabrication of engineered materials. The use of isotope exchange‐atom probe tomography allowed precise overlap of the oxygen diffusion pathways at the nanoscale and the 3D reconstruction of the cationic distribution, which appears to be highly inhomogeneous for Sr. Dopant and oxygen vacancy accumulation areas are spatially separate and the resulting ionic defect distribution is strongly asymmetric around the grain boundaries. We propose that the use of atom probe tomography coupled to oxygen isotope exchange is a widely applicable technique for the study of local mass transport phenomena which allows obtaining previously undisclosed insights into the grain boundary structure and chemical environment.

## 4 Experimental Section

### Thin‐Film Deposition

Thin films of La_0.9_Sr_0.1_CrO_3_ were fabricated by large‐area pulsed laser deposition (PVD Systems—PLD 5000) equipped with a 248 nm KrF excimer laser (Lambda Physics—COMPex PRO 205) under the following conditions: oxygen pressure 6.6 × 10^–3^ mbar, target‐substrate distance 90 mm, laser fluency ≈1.1 J cm^–2^. The nanocrystalline samples were deposited at 750 °C with a laser repetition rate of 10 Hz and using an Al_2_O_3_ (0001) single crystal substrate (Crystec GmbH). An interlayer of Ce_0.8_Gd_0.2_O_1.9_ was deposited on top of the alumina substrate under the same conditions. The epitaxial samples were deposited at 800 °C with a laser repetition rate of 2 Hz and using a SrTiO_3_ (001) single crystal substrate (Crystec GmbH). A commercial target was employed.

### Global Structural Characterization

X‐ray diffraction (XRD) was carried out using PANalytical X'Pert‐PRO MRD diffractometer. Full scans (10°–130°) were measured using a parabolic mirror (incident radiation Cu_kα1_ = 1.54060 Å, Cu_kα2_ = 1.54443 Å) and Ni filter. The step‐size was 0.001° and counting time 25 s. For high‐resolution XRD and reciprocal space mapping (RSM), an asymmetric 4‐reflections Ge220 monochromator, combined with a parabolic mirror, was employed (incident radiations Cu_kα1_). 2θ–ω HR scan employs a step‐size of 0.005° and counting time 1 s. For RSM, a step size of 0.01° for ω and 2θ (counting time 20 s) was used. Atom force microscopy (AFM) was carried out in a Park System and analyzed by Gwyddion software.

### Isotope Exchange and ToF‐SIMS

LSCr thin‐film sample surfaces were cleaned by pure ethanol and acetone before the oxygen isotope exchange. First, an annealing in 200 mbar of pure oxygen N5 (99.999%) with ^18^O_2_ in the normal isotopic abundance was performed. The exchanged tube was then pumped down and filled with an ≈90% ^18^O_2_ enriched gas (200 mbar). After the exchange, the sample was quenched to room temperature. The exchange temperature and time were 639 °C and 30 min, respectively. The LSCr–STO and LSCr/CGO/Al_2_O_3_ samples were treated at the same time to reduce the experimental error. Once exchanged, the ^18^O‐ diffusion profile signal was recorded using a TOF‐SIMS 5 instrument (ION‐TOF GmbH, Munster, Germany). The negative ion analysis was performed using a liquid metal bismuth gun (LMIG). A 25 keV Bi^+^ primary ion beam was rastered on the sample surface to generate the secondary ion detected in the burst alignment mode. A secondary Cs^+^ ion beam (2 keV) was employed for the depth profile analysis. The analysis beam area was 50 µm × 50 µm while the sputtering area was 150 µm × 150 µm.

### Transmission Electron Microscopy

A plane‐view thin lamella for transmission electron microscopy (TEM) was prepared by mechanical grinding and polishing parallel to the sample surface, followed by ion milling to reach electron transparency. The high‐resolution TEM images were obtained in a JEOL 2010F TEM, working at 200 kV acceleration voltage.

### Atom Probe Tomography

Atom probe tomography (APT) specimen preparation was carried out on a FEI Helios NanoLab 600i focused ion beam/scanning electron microscope (FIB/SEM) or a Tescan S8252G Raman imaging FIBs‐1EM. In both cases, specimens were prepared using a lift‐out technique where five needle specimens were fabricated from a single lift‐out section and were mounted on TEM grids held by hardware that allowed for TEM imaging (FEI Talos F200X) and analysis of the APT specimens refs. ^[^ ^54^, ^55^ ^]^. Initial shaping was performed using a 30 kV Ga^+^ ion beam voltage.  After initial imaging in the TEM, some specimens were further sharpened in the FIB using a 2 kV ion beam voltage to bring the region of interest closer to the specimen apex. The total FIB preparation time per set of five specimens was about 3 h. The three best specimens from each sample, as determined from TEM imaging, were analyzed by APT.

APT (Cameca LEAP 4000X Si) was performed at 45.5 K using a 20 pJ laser energy and 350–500 kHz pulse rates. The flight path length was 90 mm and the ion detection rate was set to 5 ions per 1000 pulses, resulting in a bias range of 2000–6000 V during the data collection.  Reconstructions were generated in Cameca's IVAS 3.6.18 software using the TEM images of the specimens and the method of ref. ^[^ ^56^ ^]^ for setting the reconstruction parameters.  A systematic energy deficit correction was employed to improve the mass spectral resolution ref. [^57^] Two of the three analyzed epitaxial LSCr APT specimens produced usable data (one fractured early in the data collection); and all three of the analyzed nanocrystalline LSCr APT specimens produced good data.  The actual APT data collection time (ignoring set up and laser alignment) took between 15 and 90 min per specimen.

## Conflict of Interest

The authors declare no conflict of interest.

## Supporting information

Supporting Information

Supplemental Video 1

## Data Availability

The data that support the findings of this study are openly available in Zenodo at https://doi.org/10.5281/zenodo.5245171.
