# Supplementary material for: Direct Measurement of Oxygen Mass Transport at the Nanoscale
Source: Adv Mater. 2021 Oct 5;33(48):2105622. doi: 10.1002/adma.202105622 (PMC11468452; doi:10.1002/adma.202105622)
Supplement: Supplementary file 1 — Supporting Information [file ADMA-33-2105622-s002.pdf]

© 2021 Wiley-VCH GmbH

ADVANCED MATERIALS

Supporting Information

for *Adv. Mater.,* DOI: 10.1002/adma.202105622

Direct Measurement of Oxygen Mass Transport at the Nanoscale

*Federico Baiutti, Francesco Chiabrera, David Diercks, Andrea Cavallaro, Lluís Yedra, Lluís López-Conesa, Sonia Estradé, Francesca Peiró, Alex Morata, Ainara Aguadero, and Albert Tarancón**

Supporting Information

Direct measurement of oxygen mass transport at the nanoscale

*Federico Baiutti,† Francesco Chiabrera,† David Diercks, Andrea Cavallaro, Lluís Yedra, Lluís López-Conesa, Sonia Estradé, Francesca Peiró, Alex Morata, Ainara Aguadero, Albert Tarancón**

† Equal contribution

Note S1. Structural characterization of $La_{0.8}Sr_{0.2}CrO_3$ thin films

The microstructural characterization of $La_{0.8}Sr_{0.2}CrO_3$ (LSCr) thin films deposited on 130 nm CGO/Al_2O_3 (0001) reveals a polycrystalline structure of both LSCr and CGO layers, as shown in the X-Ray diffraction (XRD) patterns of Supplementary Figures 1a-c. All the diffraction peaks were assigned to the pseudo-cubic unit cell of LSCr and CGO. The main orientations for LSCr are (001) and (011); Note that an additional diffraction peak from (111) orientation may superimpose with the substrate peak. Supplementary Figure 1d shows the atomic force microscope (AFM) image of the LSCr surface. A fully dense nanocrystalline structure is observed, with an average grain size of 35 nm. Supplementary Figure 2a shows the theta-2theta scan of the LSCr thin films deposited on $SrTiO_3$ (001). The XRD pattern shows a highly oriented (001) LSCr layers. High resolution XRD around the 001 peaks reveals the presence of finite-size oscillations, indicative of a long-range homogeneity of the film (see Supplementary Figure 2b). The Reciprocal Space Maps (RSM) around the 204 peak of the substrate shows a very good match between the in-plane and out-of-plane lattice parameters of the film and the substrates (Supplementary Figure 2c), revealing a fully strained epitaxial growth of the LSCr layers. Finally, a low surface roughness of 0.6 nm is measured by AFM in Supplementary Figure 2d.

Transmission Electron Microscopy (TEM) images are reported in Supplementary Figure 3. They highlight a dense structure with grains around 34 nm in diameter (measured in Supplementary Figure 3a). Grain sizes and morphology fit well with the observed diffusion channels in APT. Fast Fourier Transforms (FFTs) of the HRTEM images allow for indexation of the growth direction, which could be assigned most regularly to [001] or [111] pseudocubic directions (Supplementary Figures 3b to e). A certain degree of mosaicity (measured at 6.5˚) is observed in the FFTs. Overlapping boundaries in panels b, d show moiré patterns arising from the relative crystal rotation between the grains.

Note S2. Fitting of oxygen mass transport parameters in epitaxial $La_{0.8}Sr_{0.2}CrO_3$ thin films

The oxygen mass transport parameters of epitaxial LSCr (001) thin films were obtained by fitting the isotope fraction with the semi-infinite solution of oxygen diffusion:[1]

$$f(^{18}O) = c_{bg} + \left(c_g - c_{bg}\right) * \left(erfc\left(\frac{z}{2\sqrt{D^*t}}\right) - \left(\exp(hz + h^2D^*t\right) * erfc\left(\frac{z}{2\sqrt{D^*t}} + h\sqrt{D^*t}\right)\right) \qquad \text{Supplementary Eq. 1}$$

Where c_{bg} and c_g are respectively the natural isotope background concentration (0.2%) and the isotope enriched gas concentration (90%), t is the exchange time and h is equal to D*/k*. Supplementary Figure 4 shows depth profile of the isotope fraction measured in the epitaxial layer along with the curve fit obtained with Supplementary Equation 1. The figure shows a good agreement between experimental results and analytical model. The oxygen mass transport parameters extracted by the analysis are:

$D^*_b = 2.0 \cdot 10^{-16}$ cm^2/s and $k^*_b = 2.0 \cdot 10^{-16}$ cm^2/s.

Note S3. 3D Finite Element Model of APT oxygen transport in $La_{0.8}Sr_{0.2}CrO_3$ thin films

Finite Element Method (FEM) was used to model ^{18}O diffusion and incorporation in LSCr polycrystalline thin films. The simulations were performed by COMSOL Multiphysics using the *Transport of Diluted Species* module, which solves Fick's law. Supplementary Figure 5 shows the 3-dimensional geometry used in the simulations. The dimensions and shape of the LSCr grains were directly modelled on the 3D renderings of tracer fraction measured by APT, while a width of one nm was considered for the GB regions. The thickness of the LSCr and the CGO layer was set to reproduce IEDP-SIMS and APT measurements (45 nm and 130 nm, respectively). Supplementary Figure 6 schematically shows the oxygen mass transport parameters and the boundary conditions used in the model. Two different diffusivity coefficients, D^*_b and D^*_{gb}, were considered for bulk and GB domains, respectively. On the top surface, a convective type boundary equation was set for simulating oxygen incorporation, while zero flux was imposed on all other boundary surfaces. In agreement with previous works,[2–4] two different oxygen incorporation parameters were set for bulk and grain boundary exposed surfaces (k^*_b and k^*_{gb}). A uniform initial tracer fraction of 0.002 was set to simulate the natural isotope background concentration. Time dependent Fick´s second law of diffusion was then numerically solved for a time equal to the ^{18}O exchange annealing (1803 s).

The simulations were performed in an iterative fashion in order to find the set of oxygen transport parameters that better resemble the APT measurements. In this sense, 1D ^{18}O tracer profiles of bulk and GB regions were used to speed up the process. Supplementary Figure 7 shows a set of different horizontal 1D tracer profiles complementary to the ones shown in Figure 1 of the main text. A good match between experimental data and simulations is observed.

Note S4. 2D equivalent Model of SIMS oxygen transport in $La_{0.8}Sr_{0.2}CrO_3$ thin films: SIMS fitting

A 2D FEM model was developed for fitting the IEDP-SIMS depth profiles with COMSOL Multiphysics. The model´s geometry is based on the use of an equivalent grain and GB dimension, similar to previous works.[3,4] The grain dimension was set to 35 nm while the GB to 1 nm. The FEM model uses the same boundary conditions, physics and initial concentration described before for the 3D FEM model. Two different oxygen diffusivities and surface exchange coefficients were used as free parameters, k_{gb}, D_{gb} and k_b, D_b for GBs and bulk, respectively. These parameters were varied to obtain an integrated tracer fraction similar to the one measured by IEDP-SIMS.

Supplementary Figure 8 shows that two different sets of oxygen transport parameters (named FEM1 and FEM2) lead to a tracer depth profile similar to the one measured by IEDP-SIMS. These sets of parameters are characterized by almost one order of magnitude difference in k_{gb}, D_{gb}, k_b and D_b as shown in Supplementary Table 1. Supplementary Figures 8b and c show the ^{18}O fraction 2D maps obtained for FEM1 and FEM2, respectively. In FEM2, the higher k_{gb} and lower D_{gb} determines an increase of $f(^{18}O)$ at the GBs, reaching concentrations around 0.8. On the other hand, FEM1 presents ^{18}O fraction of about 0.38 in the GBs, similar to the one measured by IE-APT. Indeed, as shown in Supplementary Table 1, the oxygen mass transport parameters of FEM1 are similar to the ones obtained by 3D FEM modelling of IE-APT data. A certain discrepancy is observed in the bulk parameters, probably due to the large inspection area of IEDP-SIMS and to the approximation of an equivalent grain dimension, which does not take into account grain size distribution and other types of 3D effects. Overall, the analysis highlights the great advantages of IE-APT in precisely determining the oxygen mass transport coefficients in the case of heterogeneous diffusion and oxygen incorporation.

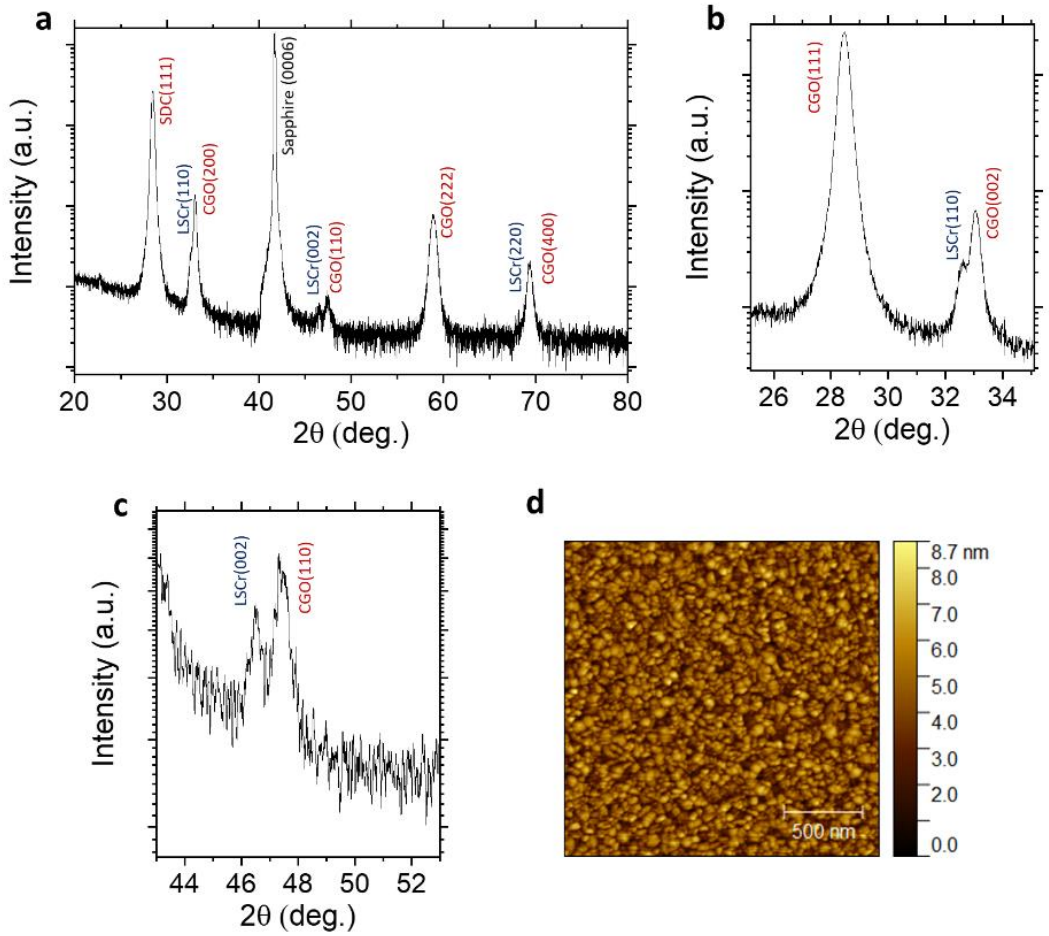

Figure S1. Structural characterization of polycrystalline LSCr thin films. a) Theta-2theta XRD pattern and magnification of two different regions (b and c). d) AFM image of the sample surface

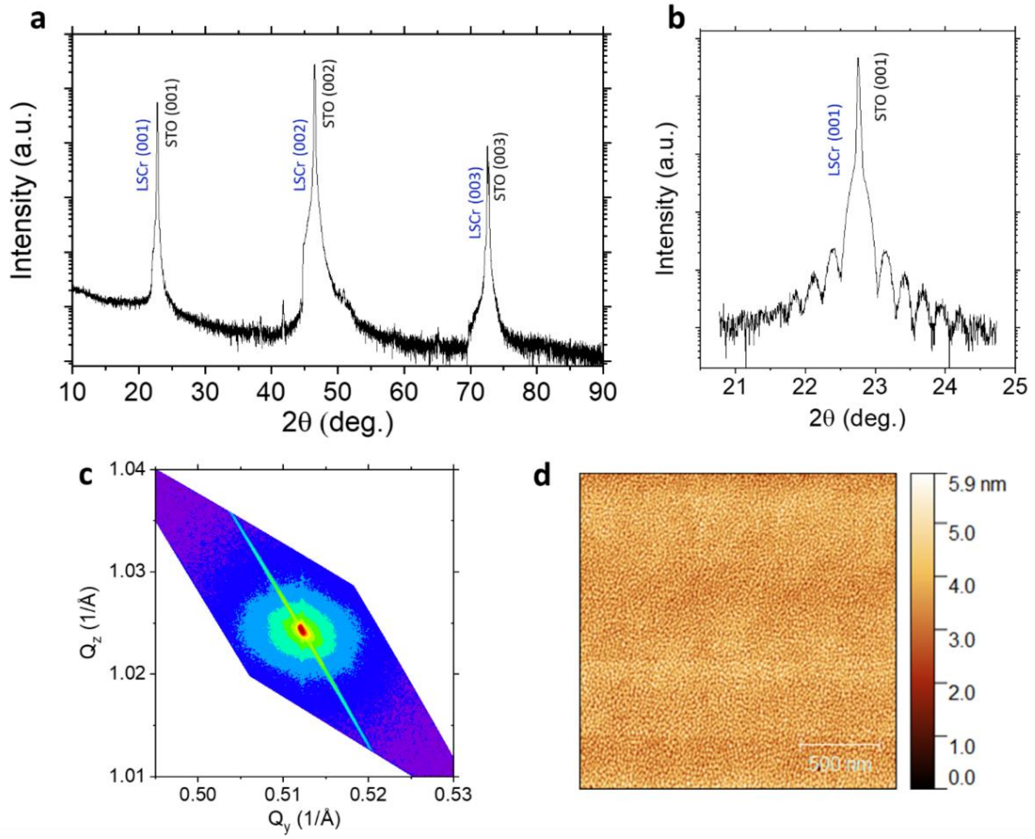

Figure S2. Structural characterization of epitaxial LSCr thin films. a) Theta-2Theta scans of the LSCr thin film on $SrTiO_3$. b) high resolution theta-2theta scan around the 001 substrate peak. c) RSM of the 204 asymmetrical refraction. d) AFM image of the sample's surface

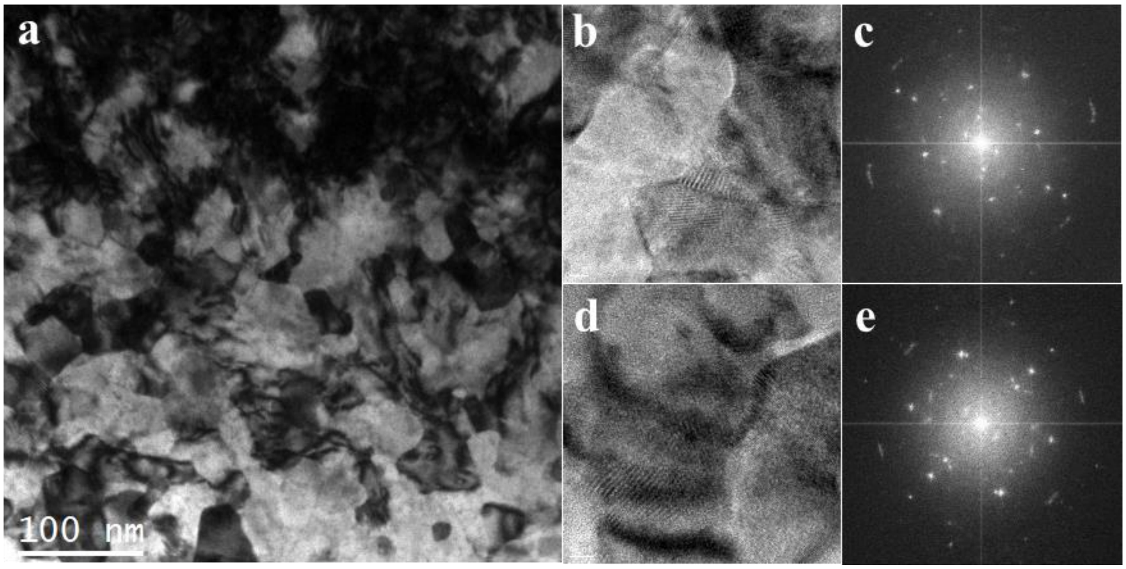

Figure S3. (a) Bright Field TEM image from which an average grain size of 34 nm was measured. (b), (d) HRTEM images with corresponding FFTs in (c) and (e).

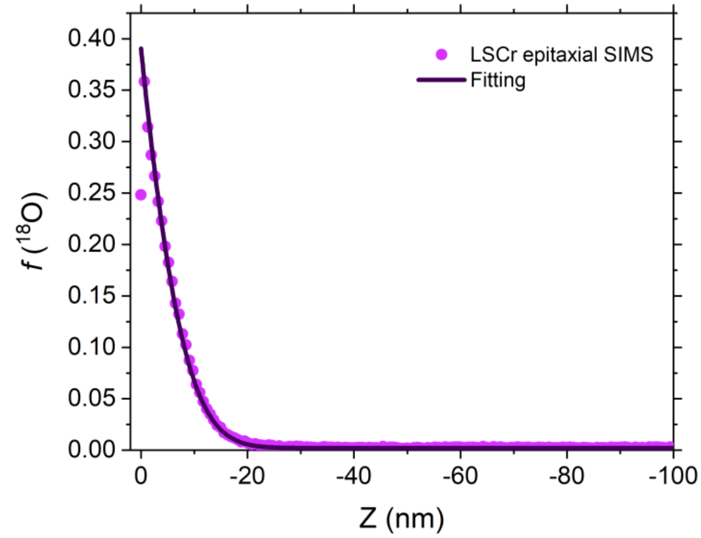

Figure S4. Depth profiles of the ^{18}O fraction measured by IEDP-SIMS in LSCr epitaxial thin films annealed at 640 °C for 30 min in a 90% ^{18}O enriched atmosphere, together with the curve fit.

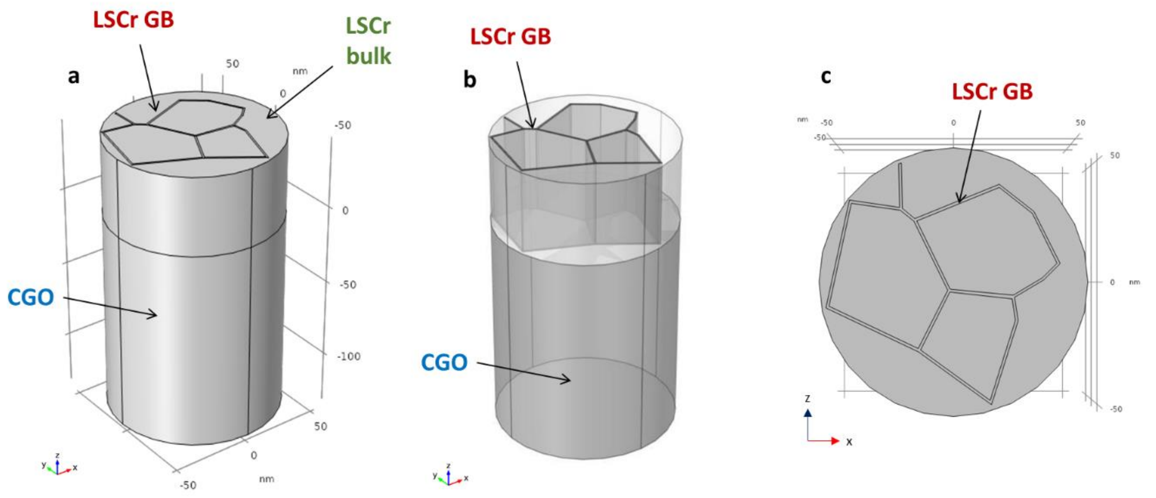

Figure S5. a) and b) 3D geometry used in the FEM simulations to model ^{18}O fraction obtained by APT. c) Top view of the 3D geometry showing the grain and GBs shapes.

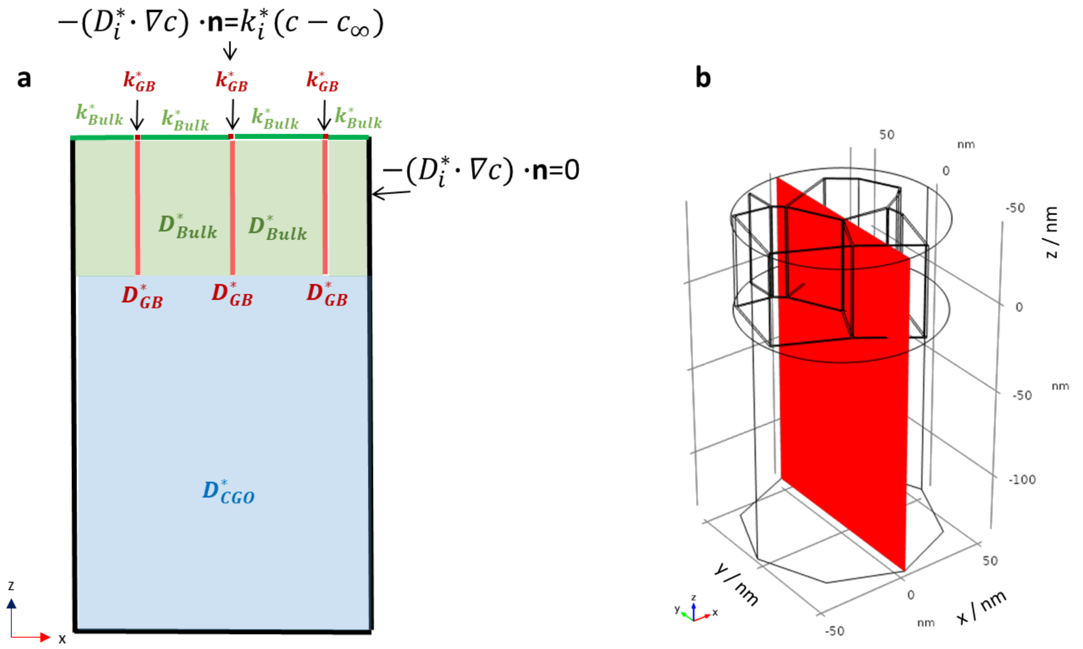

Figure S6. a) Sketch of the cross sectional area shown in red in (b), showing the main oxygen transport parameters and the boundary conditions considered in the FEM model.

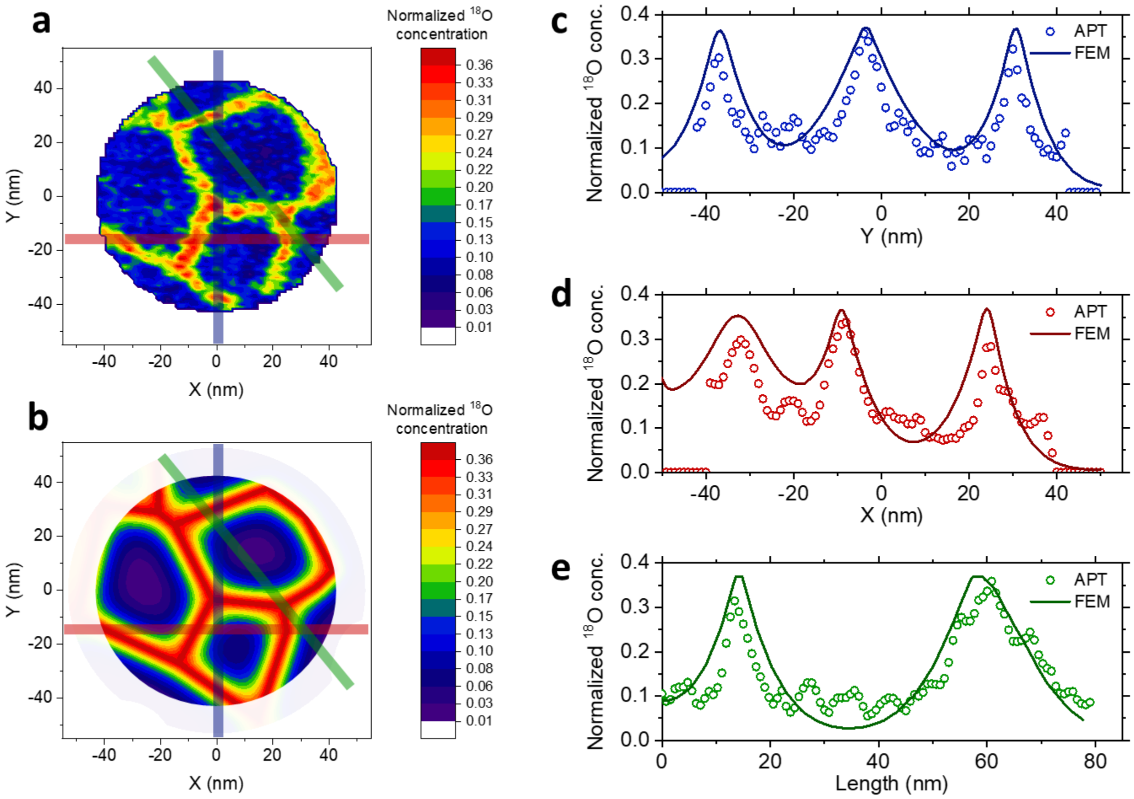

Figure S7. Horizontal cross section obtained from IE-APT measurements (a) and FEM simulations (b) at z = -27 nm from the surface. c-e) 1D tracer profiles extracted from the line cuts shown in (a) and (b).

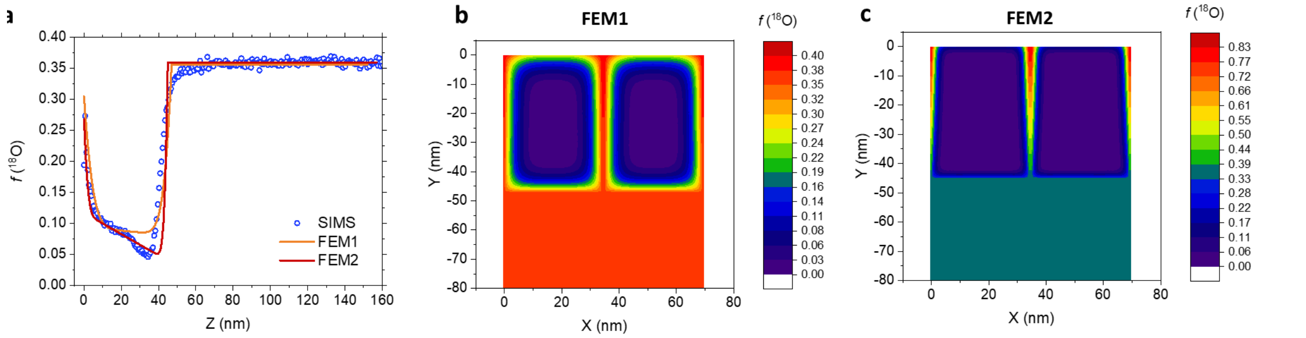

Figure S8. a) Depth profiles of ^{18}O fraction measured by IEDP-SIMS for the polycrystalline LSCr. The best fitting obtained with model FEM1 and FEM2 are also shown in a. 2D ^{18}O vertical maps obtained in FEM1 (b) and FEM2 (c). Despite the large differences of concentration locally calculated, the two models give rise to similar integrated $\boldsymbol{f(^{18}O)}$ depth profiles. FEM1 provides the better match to the IE-APT results.

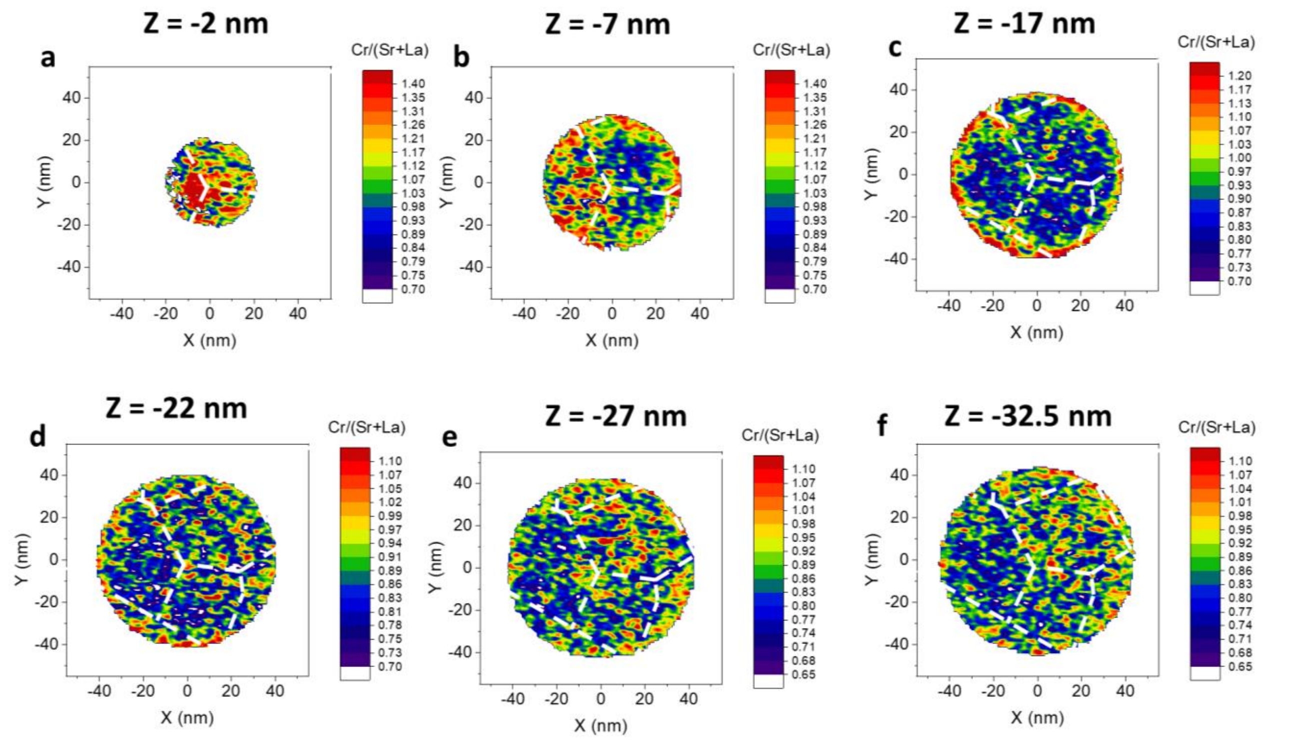

Figure S9. IE-APT results for the Cr/(Sr+La) (B/A) ratio for different in-plane contour plots. The dashed lines indicate the GB locations.

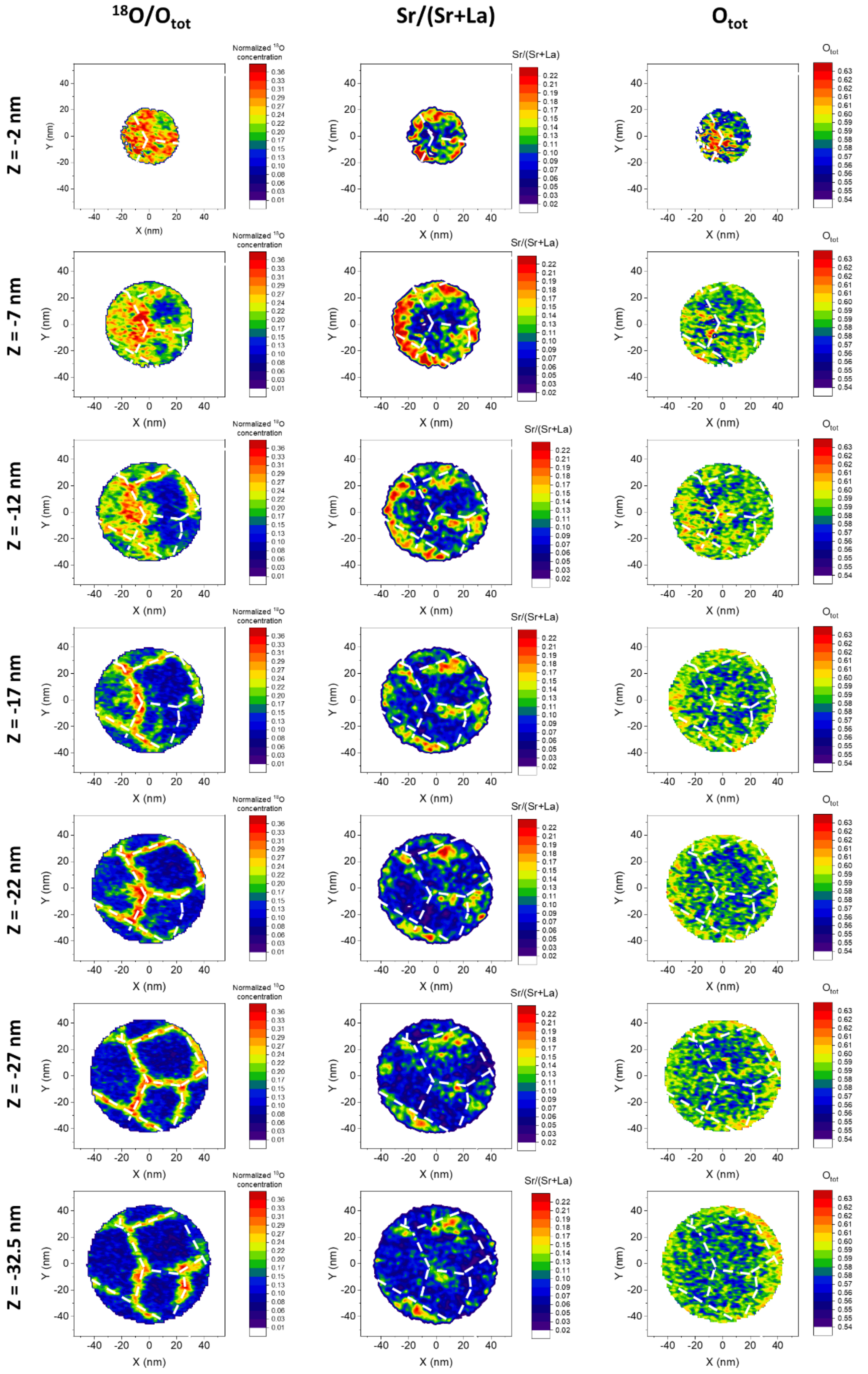

Figure S10. Comparison between oxygen isotope, Sr, and total oxygen distributions from the IE-APT results. The dashed lines indicate the GB locations

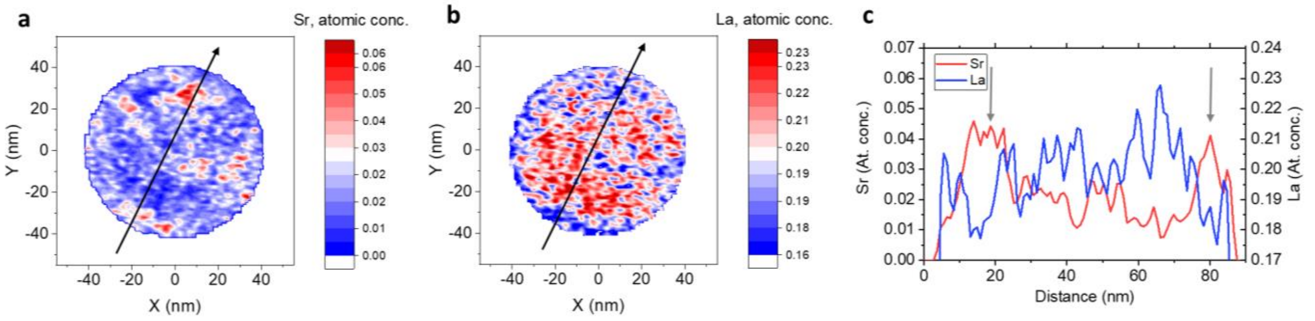

Figure S11. Sr (a) and La (b) atomic concentrations by APT (z =-22 nm). c) Concentration profile along the line shown in panels (a) and (b).

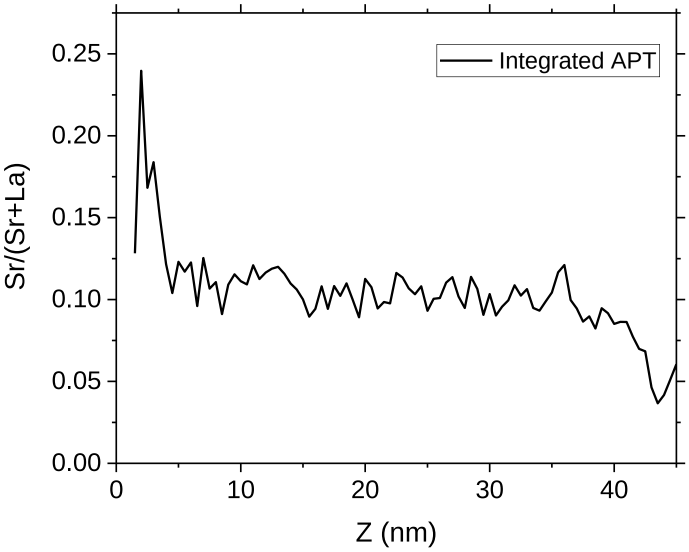

Figure S12. Out-of-plane Sr concentration profile, integrated over the vertical contour plot reported in Fig. 4 of the main text.

Table S1. Oxygen transport parameters obtained by FEM modelling of the IEDP-SIMS tracer fraction according to model FEM1 and model FEM2. The coefficients obtained by the IE-APT analysis and in the epitaxial GB-free sample are also reported for comparison.

	D^*_b (cm²/s)	D^*_{gb} (cm²/s)	k^*_b (cm/s)	k^*_{gb} (cm/s)
Poly APT-3D FEM	$2.5 \cdot 10^{-16}$	$6.5 \cdot 10^{-12}$	$1.5 \cdot 10^{-10}$	$9.5 \cdot 10^{-8}$
Epitaxial SIMS	$2.0 \cdot 10^{-16}$	-	$2.0 \cdot 10^{-10}$	-
Poly SIMS-2D FEM1	$1.0 \cdot 10^{-16}$	$7.0 \cdot 10^{-12}$	$8.0 \cdot 10^{-11}$	$1.2 \cdot 10^{-7}$
Poly SIMS-2D FEM2	$1.7 \cdot 10^{-17}$	$6.3 \cdot 10^{-13}$	$2 \cdot 10^{-11}$	$9 \cdot 10^{-7}$

Additional references

1. Crank, J. *The mathematics of diffusion*. (Oxford science, 1956).
2. Saranya, A. M. A. M. *et al.* Unveiling the Outstanding Oxygen Mass Transport Properties of Mn-Rich Perovskites in Grain Boundary-Dominated $La_{0.8}Sr_{0.2}(Mn_{1-x}Co_x)_{0.85}O_{3\pm\delta}$ Nanostructures. *Chem. Mater.* **30**, 5621–5629 (2018).
3. Navickas, E. *et al.* Dislocations Accelerate Oxygen Ion Diffusion in $La_{0.8}Sr_{0.2}MnO_3$ Epitaxial Thin Films. *ACS Nano* **11**, 11475–11487 (2017).
4. Navickas, E. *et al.* Fast oxygen exchange and diffusion kinetics of grain boundaries in Sr-doped $LaMnO_3$ thin films. *Phys. Chem. Chem. Phys.* **17**, 7659–7669 (2015).
